# Clinical Outcomes of Steep-Axis One-Handed Phacoemulsification under the Guidance of a Verion Image-Guided System

**DOI:** 10.1155/2019/7182324

**Published:** 2019-09-15

**Authors:** Panpan Li, Yuanyuan Tu, Xiang Chen, Yu Song, Huaijin Guan

**Affiliations:** ^1^Department of Ophthalmology, Nantong City No. 1 People's Hospital and Second Affiliated Hospital of Nantong University, Nantong 226000, Jiangsu Province, China; ^2^Eye Institute, Affiliated Hospital of Nantong University, Nantong 226001, Jiangsu Province, China; ^3^Clinical Laboratory, Nantong City No. 1 People's Hospital and Second Affiliated Hospital of Nantong University, Nantong 226000, Jiangsu Province, China

## Abstract

**Purpose:**

To compare the efficiency and safety of steep-axis one-handed phacoemulsification with steep-axis two-handed phacoemulsification.

**Patients and Methods:**

Patients with cataracts underwent steep-axis one-handed (steep-axis one-handed group) or steep-axis two-handed (steep-axis two-handed group) phacoemulsification with a 2.4 mm clear corneal incision (CCI) under the guidance of the Verion Image-Guided System. Intraoperative phacoparameters, such as visual acuity, surgically induced astigmatism (SIA), total corneal astigmatism (TCA), angle of error (AE), corneal volume (CV), and corneal endothelial cells, were compared.

**Results:**

There were no significant differences in the intraoperative phacoparameters between the two groups. The visual outcomes were significantly better in the steep-axis one-handed group than in the two-handed group at 1 week postoperatively (all *p* < 0.05) but not at 1 month and 3 months postoperatively. TCAs were significantly decreased in both groups at 1 and 3 months postoperatively (all *p* < 0.05). There were no significant differences at any follow-up points in both groups (all *p* < 0.001). At 3 months postoperatively, the SIA was 0.95 ± 0.44 D in the steep-axis one-handed group and 1.01 ± 0.50 D in the steep-axis two-handed group; there was no significant difference between the groups. The AE was 39.45 ± 26.53° in the steep-axis one-handed group and 49.75° ± 26.23° in the steep-axis two-handed group, which were significantly different (*p*=0.005). Endothelial cell loss was significantly lower in the steep-axis one-handed group than that in the steep-axis two-handed group at all follow-up points (all *p* < 0.05).

**Conclusions:**

Both the steep-axis one-handed and the steep-axis two-handed techniques could significantly decrease TCA. Compared with the steep-axis two-handed technique, the steep-axis one-handed technique has the advantage of decreasing the AE and reducing trauma to the cornea in soft-to-moderate nuclei.

## 1. Introduction

Currently, cataract surgery has entered the era of refractive surgery, and the intraoperative correction of corneal astigmatism is a necessary requirement for refractive cataract surgery (RCS). Many methods are available to correct preexisting astigmatism of the cornea, such as single clear corneal incision (CCI) or paired opposite CCI at the steepest meridian [1–4], limbal relaxing incision [5], and toric intraocular lens (IOL) implantation [6]. Precisely measuring corneal astigmatism and marking the steep axis are crucial procedures for the correction of corneal astigmatism. In previous practice, cataract surgeons would usually design steep-axis CCI according to anterior astigmatism (AA) or keratometric astigmatism (KA) because instruments to measure total corneal astigmatism (TCA) were not commercially available. Surgeons marked the steep axis of corneal astigmatism using manual marking techniques under direct contact microscopy without a digital navigation system. Pentacam was based on the Scheimpflug principle and measured both the anterior and posterior corneal surfaces. The device measures TCA by a ray tracing technique. The Verion Image-Guided System was introduced to provide automated tracking. This system has several components, including the measurement module, the vision planner, and the digital markers microscope, and laser. The image-guided planning system provides integrated digital guidance for the alignment of the corneal incision and the toric IOL axis [7].

Currently, there are two kinds of phacoemulsification: one-handed phacoemulsification and two-handed phacoemulsification. Our previous studies have found that the one-handed technique had the advantage of reduced trauma to the cornea compared with the two-handed technique for cataract patients with a soft-to-moderate nucleus [8, 9]. The main objective of this study was to compare the efficiency and safety of steep-axis (based on the steep meridian of TCA) one-handed phacoemulsification with steep-axis two-handed phacoemulsification under the guidance of the Verion Image-Guided System.

## 2. Patients and Methods

This prospective nonrandomized comparative study comprised patients with age-related cataract (ARC) who received phacoemulsification at the Department of Ophthalmology, Affiliated Hospital of Nantong University, Jiangsu, China, from September 2017 to May 2018. All patients agreed to participate, met the inclusion criteria, chose a surgical technique according to the patient's wishes, and signed an informed consent agreement before undergoing any procedure, and the eye conditions were matched between two groups. The study was performed in accordance with the ethical principles of the Declaration of Helsinki and approved by the Affiliated Hospital of Nantong University's ethics committee.

The Lens Opacities Classification System III (LOCS III) was used for cataract classification [10]. Inclusion criteria included the following: (1) patients with ARC and regular corneal astigmatism, (2) TCA>0.5 D, (3) nuclear opalescence (NO) ≤3, (4) normal anterior segment and fundus examination, and (5) no history of intraocular surgery or injury. Exclusion criteria included the following: (1) cataract other than ARC and (2) corneal scars or opacities and other ocular disease that might affect visual outcomes. Patients undergoing phacoemulsification were divided into two groups based on the type of surgical technique: steep-axis one-handed technique with a 2.4 mm CCI created at the steep axis of the cornea (steep-axis one-handed group) or steep-axis two-handed technique with a 2.4 mm CCI created at the steep axis of the cornea and a 1.0 mm cornea side incision set at the limbus far from CCI at approximately 115° (steep-axis two-handed group). Eye conditions, such as axial length, white-to-white (WTW), central corneal thickness (CCT), anterior chamber depth (ACD), NO, lens thickness, and intraocular pressure (IOP), were matched between two groups.

### 2.1. Preoperative Measurements

All patients underwent a complete ophthalmologic measurement. Visual acuity included uncorrected and corrected distance visual acuity (UDVA and CDVA) and uncorrected and corrected near visual acuity (UNVA and CNVA). Biometry, such as axial length, WTW, and CCT, was performed using a Lenstar noncontact optical low-coherence reflectometer (LS900; Haag-Streit, Koniz, Switzerland). Corneal volume (CV) and TCA were obtained using a Pentacam (70700, Oculus, Wetzlar, Germany). Anatomic landmarks of the eye were obtained using the Verion Image-Guided System (Alcon Laboratories, Inc., Fort Worth, TX, America).

### 2.2. Surgical Planning

**Figure 1:**
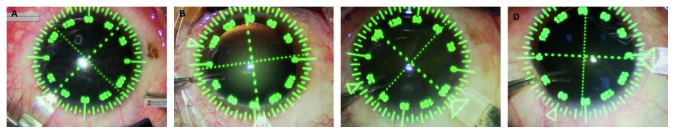
The 2.4 mm steep-axis clear corneal incision (CCI) was created at different positions under the Guidance of Verion Image-Guided System. The steep-axis CCI was created at (a) 50°, (b) 95°, (c) 145°, and (d) 175°.

The anatomic image was transferred onto a USB drive inserted into the Verion digital marker monitor screen, which was used to guide the different positions of steep-axis CCI. The 2.4 mm CCI was selected for the steep axis, which was chosen according to the steepest corneal meridian of the TCA. The triangle representing the target incision appeared both on the monitor and through the oculars on the microscope (Figure 1).

### 2.3. Surgical Techniques

All operations were performed under topical anesthesia with 0.5% proparacaine hydrochloride (Novartis, Switzerland) by the same experienced surgeon (H.G.). Pupillary dilation was achieved with the instillation of one drop of 0.5% compound tropicamide (Santen, Japan) every 10 minutes until the pupil diameter was greater than 7 mm before surgery. In all cases, sodium hyaluronate gel (Bausch & Lomb, Bridgewater, America) was used as an ophthalmic viscosurgical device (OVD) and balanced salt solution (BSS) as the infusion fluid. All phacoemulsifications were performed using a phacoemulsification machine (Centurion, Alcon, Fort Worth, America). Phacoemulsification was performed using OZil Intelligent Phaco (IP) software. The ultrasound and fluid settings were as follows: bottle height: 105 cm, power: 60%, vacuum: 500 mmHg, and aspiration flow: 32 cc/min.

#### 2.3.1. Steep-Axis One-Handed Phacoemulsification

A 2.4 mm single-plane CCI was created at the steep axis of the cornea under the guidance of the Verion Image-Guided System with a diamond keratome. Continuous curvilinear capsulorhexis (CCC) was performed with capsular forceps under the protection of an OVD. After CCC, hydrodissection and hydrodelineation were performed, and nuclear emulsification was performed using the one-handed technique [8]. All phacoemulsification was performed using the phacoemulsification machine (Centurion, Alcon, Fort Worth, America) in the burst mode. A foldable aspheric surface monofocal IOL was implanted in the capsular bag with an injector. At the end of the surgery, the clear corneal wound was hydrated with a balanced salt solution, and no sutures were applied.

#### 2.3.2. Steep-Axis Two-Handed Phacoemulsification

A 2.4 mm single-plane CCI was created at the steep axis of the cornea, and a 1.0 mm cornea side incision was set at the limbus far from CCI (at approximately 115°) in the two-handed group, under the guidance of the Verion Image-Guided System. CCC was performed with capsular forceps under protection of an OVD. After CCC, hydrodissection and hydrodelineation were performed, and nuclear emulsification was performed using the two-handed technique [8]. The rest of the procedure was similar to that used in the one-handed technique.

#### 2.3.3. Intraoperative Phacoparameters

The following intraoperative phacoparameters were recorded: cumulative dissipated energy (CDE), ultrasonic total time, and total surgical time (the time between the creation and closure of the corneal incision by stromal hydration).

### 2.4. Postoperative Parameters

All patients underwent ophthalmologic measurements at follow-up appointments: 1 week, 1 month, and 3 months postoperatively. Visual acuity included uncorrected and corrected distance visual acuity (UDVA and CDVA) and uncorrected and corrected near visual acuity (UNVA and CNVA), CV within the central 10 mm zone (10-mm CV), TCA, and central endothelial cell measurements. SIA and angle of error (AE) at 3 months postoperatively were analyzed by vector analysis according to the Alpins method [11].

### 2.5. Statistical Analysis

Statistical analysis was performed using SPSS for Windows software (version 22, SPSS, Inc). The homoscedasticity of the data was studied with Levene's test. For the comparison of steep-axis one-handed and steep-axis two-handed group data, Student's *t*-tests for two independent samples were used. The chi-square test was used for comparison of the type of corneal astigmatism. For the comparison of preoperative and postoperative data, Student's *t*-test of paired samples was used. The level of statistical significance was always *p* < 0.05.

## 3. Results

The study evaluated 209 eyes of 185 patients: 104 eyes of 96 patients in the steep-axis one-handed group and 105 eyes of 96 patients in the steep-axis two-handed group. Seven patients underwent steep-axis one-handed phacoemulsification in one eye and two-handed phacoemulsification in the other eye. Patients' demographics and preoperative ophthalmic measurements are provided in Table 1. No significant differences in age, axial length, WTW, CCT, ACD, NO, lens thickness, IOP, and types of TCA were noted between the 2 groups (all *p* > 0.05).

The surgical parameters in both groups are summarized in Table 2. The CDE, ultrasound total time, and total surgical time was 5.57 ± 4.19, 30.72 ± 23.13 s, and 353.77 ± 59.86 s, respectively, in the steep-axis one-handed group and 5.29 ± 4.27, 26.23 ± 20.28 s, and 350.07 ± 50.79 s, respectively, in the steep-axis two-handed group. No significant differences were noted between the 2 groups (all *p* > 0.05).

Table 3 displays the visual acuity, TCA, SIA, and angle of error (AE) between the 2 groups. Compared with baseline, UDVA, CDVA, UNVA, and CNVA were significantly improved at all follow-up points in both groups (all *p* < 0.001). At 1 week postoperatively, UDVA, CDVA, UNVA, and CNVA in the steep-axis one-handed group were significantly improved compared with those in the steep-axis two-handed group (all *p* < 0.05). However, the abovementioned differences were ameliorated at 1 and 3 months postoperatively (all *p* > 0.05). Compared with baseline, TCAs were improved at 1 week postoperatively in both groups, but there were no significant differences (all *p* > 0.05). At 1 and 3 months postoperatively, TCAs were significantly decreased in both groups (all *p* < 0.05). There were no significant differences in TCA at all follow-up points in both groups (all *p* < 0.001). At 3 months postoperatively, the SIA was 0.95 ± 0.44 D in the steep-axis one-handed group and 1.01 ± 0.50 D in the steep-axis two-handed group; this difference was not statistically significant (*p*=0.363). The AE was 39.45° ± 26.53° in the steep-axis one-handed group and 49.75 ± 26.23° in the steep-axis two-handed group, which was a significant difference (*p*=0.005).

Table 4 presents the 10 mm CV and central endothelial cell data of the 2 groups. In both groups, the mean 10 mm CV was significantly increased at 1 week (all *p* < 0.001) and 1 month postoperatively (all *p* < 0.05); the values returned to preoperative levels at 3 months postoperatively (all *p* > 0.05). The between-group difference was statistically significant at 1 week postoperatively (*p*=0.005) but not at 1 month (*p*=0.467) or 3 months postoperatively (*p*=0.736). In both groups, the mean ECD was significantly decreased postoperatively (all *p* < 0.001), and no significant difference was observed between the 2 groups at any follow-up points (all *p* > 0.05). Endothelial cell loss (ECL) was significantly lower in the steep-axis one-handed group than in the steep-axis two-handed group at all follow-up points (all *p* < 0.05). In both groups, the mean percentages of hexagonal cell (HEX%) were significantly decreased postoperatively (all *p* < 0.005), and no significant differences were observed between the 2 groups at any follow-up points (all *p* > 0.05).

In the steep-axis one-handed phacoemulsification group, the CCT was 525.45 ± 31.24 *μ*m (range: 449 to 619 *μ*m), the WTW was 11.59 ± 0.31 mm, (range: 10.86 to 12.32 mm), and the SIA at 3 months postoperatively was 0.95 ± 0.44 D. There was no significant correlation between CCT and SIA (*r* = −0.54; *p*=0.585) but significant negative correlation between WTW and SIA (*r* = −0.321; *p*=0.001). In the steep-axis one-handed phacoemulsification group, the CCT was 530.13 ± 32.27 *μ*m (range: 482 to 615 *μ*m), and the WTW was 11.61 ± 0.32 mm (range: 10.90 to 12.38 mm) The SIA at 3 months postoperatively was 1.01 ± 0.50 D. There was significant negative correlation between CCT and SIA (*r* = −0.265; *p*=0.06) but no significant correlation between WTW and SIA at 3 months (*r* = −0.102; *p*=0.303).

## 4. Discussion

Improvements in surgical techniques and instruments have propelled CCI into the current trend of phacoemulsification [8]. In RCS, CCI at the steepest meridian is the simplest and most commonly used surgical procedure for correcting preexisting astigmatism of the cornea. The main objective of our study was to compare the efficiency and safety of steep-axis one-handed with steep-axis two-handed phacoemulsification techniques. To limit bias, patients assigned to the two groups were similar in preoperative eye characteristics. WTW, CCT, and types of TCA were associated with the correction of corneal astigmatism. ACD, NO, and lens thickness were associated with intraoperative corneal injury.

The efficiency of both techniques was evaluated by the amount of CDE and total ultrasound time in two groups. In our study, the CDE and total ultrasound time were slightly higher and longer in the steep-axis one-handed group than that in the steep-axis two-handed group, but the differences were not statistically significant, which was in accordance with our previous study [8, 9]; thus, the data suggest that steep-axis one-handed phacoemulsification did not negatively affect ultrasound efficacy without the help of chop. In terms of safety, in the entire one-handed process, the phacotip was completely buried in the nucleus, which blocked the energy release and reduced heat injury. In addition, nuclear fragments and/or other mechanical trauma to the corneal endothelium were significantly decreased [8].

Previous studies hypothesized that CCI at the steep axis could flatten the steep meridian and steepen the flat meridian so as to reduce corneal astigmatism [1, 12]. Borasio et al. reported that corneal astigmatism was 1.18 ± 0.67 D preoperatively and 0.97 ± 0.54 D postoperatively in the steep-axis CCI group, and the difference was statistically significant (*p*=0.03) [1]. In our study, TCA was significantly decreased at 3 months postoperatively in both groups (*p* < 0.005).

The SIA is an integral component of refractive surgery, and personalized SIA power has been advocated in the planning of cataract surgery. SIA is influenced by the incision numbers, size (width and length), configuration (1-step, 2-step, and 3-step), and location (clear corneal on-axis and temporal incisions) [8, 12–14]. Cavallini et al. [15] reported a mean SIA value of 0.72 D one month after phacoemulsification with a 2.2 mm CCI at the 10 o'clock position and a 1.4 mm CCI at the 2 o'clock position. Kawahara et al. [12] reported that the mean SIA was 0.40 ± 0.28 D in the one-handed technique group and 0.39 ± 0.25 D in the two-handed technique group (*p*=0.84) with a 2.4 mm transconjunctival single-plane sclerocorneal incision. Koç et al. [16] reported a mean SIA value of 0.85 ± 0.42 D with a 2.8 mm superior CCI and two 1 mm side port incisions 90° from the main port. In our study, SIA was slightly higher than in previous studies, except for the effect from the surgeon. The CCT and WTW also affect SIA. Woo and Lee [17] reported that CCT was negatively correlated with the amount of SIA (in his study, CCI was 537 ± 30 *μ*m, and SIA was approximately 0.6 D with a 2.7 mm CCI). Theodoulidou et al. [18] reported that SIA was 0.77 ± 0.43 D in group A (WTW ≤ 11.6 mm), 0.69 ± 0.34 D in group B (WTW: 11.7 to 11.9 mm), 0.62 ± 0.36 D in group C (WTW 12.0 to 12.2 mm), and 0.49 ± 0.27 D in group D (WTW ≥12.3 mm) with a 3.0 mm 3-step CCI. In our study, there was no significant correlation between CCT and SIA (*r* = −0.54; *p*=0.585) but significant negative correlation between WTW and SIA (*r* = −0.321; *p*=0.001) in the steep-axis one-handed phacoemulsification group. There was significant negative correlation between CCT and SIA (*r* = −0.265; *p*=0.06), but no significant correlation between WTW and SIA at 3 months (*r* = −0.102; *p*=0.303). Our CCT was thinner than that observed by Woo and Lee [17], and our WTW was shorter than that observed by Theodoulidou et al. [18]. These differences may be the reason why our SIA is larger than that of the previous studies.

AE is the angle difference between the SIA and TIA. Positive and negative values of angle of error refer to mean counterclockwise and clockwise rotation from its intended axis, respectively [19]. The AE was significantly smaller in the steep-axis one-handed group than in the steep-axis two-handed group (*p*=0.005).

The improvement in visual acuity is an important goal of a successful cataract surgery. Visual acuity was significantly improved at all follow-up points in both groups (all *p* < 0.001). At 1 week postoperatively, visual outcomes in the steep-axis one-handed group were statistically better than those in the steep-axis two-handed group. The improvement of early visual acuity is associated with corneal trauma. Given that endothelial cellular injury alters the pumping activity of this corneal layer, resulting in increased stromal hydration [20], CV is considered as the indicative parameter of complete endothelial cell function in the area [21]. In our study, 10 mm CV was increased in the steep-axis two-handed compared with the steep-axis one-handed group at 1 week, which means corneal edema in the steep-axis two-handed group was more serious than that in the one-handed group. At 3 months postoperatively, CV in both groups was restored to previous levels, and the corneal endothelial swelling caused by phacoemulsifcation may not have lasted for 3 months. In our previous study, at 3 months postoperatively, the 10 mm CV levels were restored to the previous level after one-handed or two-handed phacoemulsifcation with a 2.4 mm CCI created at 135° position.

At present, corneal endothelial injury after phacoemulsifcation is generally assessed by specular microscopy in terms of changes in corneal endothelial cells. In our study, no significant difference in ECD was noted between the 2 groups at any follow-up point, but the mean ECL in the steep-axis one-handed group was significantly decreased compared with that in the steep-axis two-handed group. These findings were in accordance with our previous study that the one-handed technique had the advantages of less trauma to the cornea than the two-handed technique for cataract patients with a soft-to-moderate nucleus [8, 9].

In conclusion, our results indicate that both the steep-axis one-handed and the steep-axis two-handed techniques could significantly decrease TCA. Compared with the steep-axis two-handed technique, the steep-axis one-handed technique has the advantage of decreasing the AE and reducing trauma to the cornea in a soft-to-moderate nucleus.

## Figures and Tables

**Table 1:** Patient demographics and baseline parameters.

	Steep-axis one-handed	Steep-axis two-handed	*p*
Patients/eyes	96/104	96/105	—
Sex (female/male)	29/67	32/64	0.622^†^
Eye (right/left)	63/41	62/43	0.822^†^
Age (years)	66.27 ± 6.46	67.66 ± 6.16	0.113^*∗*^
Axial length (mm)	23.41 ± 1.23	23.64 ± 1.31	0.171^*∗*^
WTW (mm)	11.59 ± 0.31	11.61 ± 0.32	0.288^*∗*^
CCT (*μ*m)	525.45 ± 31.24	530.13 ± 32.27	0.762^*∗*^
ACD (mm)	3.17 ± 0.39	3.20 ± 0.36	0.586^*∗*^
NO (LOCS III)	1.98 ± 0.56	1.98 ± 0.62	0.989^*∗*^
Lens thickness (mm)	4.12 ± 0.60	4.26 ± 0.55	0.175^*∗*^
IOP (mmHg)	16.00 ± 2.08	15.77 ± 2.05	0.425^*∗*^
Type of TCA			0.553^†^
WTR (*n*)	33	36	
ATR (*n*)	34	36	
OBL (*n*)	37	33	

WTW = white-to-white; CCT = central corneal thickness; ACD = anterior chamber depth; NO = nuclear opalescence; LOCS III = Lens Opacities Classification System III; IOP = intraocular pressure; ^*∗*^independent *t*-test; ^†^Mann–Whitney *U* test; *p* < 0.05 was considered statistically significant.

**Table 2:** Intraoperative phacoparameters and total surgical time.

	Steep-axis one-handed	Steep-axis two-handed	*p*
CDE	5.57 ± 4.19	5.29 ± 4.27	0.137
U/S total time (s)	30.72 ± 23.13	26.23 ± 20.28	0.636
Total surgical time (s)	353.77 ± 59.86	350.07 ± 50.79	0.630

CDE = cumulative dissipated energy; U/S total time = ultrasound total time; *p* = comparison of both groups; *p* < 0.05 was considered statistically significant.

**Table 3:** Visual acuity and TCA.

	Steep-axis one-handed	*p* ^a^	Steep-axis two-handed	*p* ^a^	*p* ^b^
UDVA (logMAR)					
Pre-op	0.70 ± 0.14	—	0.71 ± 0.15	—	0.429
1 week post-op	0.11 ± 0.12	<0.001	0.17 ± 0.16	<0.001	0.002
1 month post-op	0.11 ± 0.10	<0.001	0.13 ± 0.10	<0.001	0.330
3 month post-op	0.12 ± 0.10	<0.001	0.12 ± 0.10	<0.001	0.948
CDVA (logMAR)					
Pre-op	0.59 ± 0.16	—	0.60 ± 0.17	—	0.766
1 week post-op	0.01 ± 0.09	<0.001	0.06 ± 0.12	<0.001	0.005
1 month post-op	0.01 ± 0.10	<0.001	0.01 ± 0.08	<0.001	0.614
3 month post-op	0.02 ± 0.10	<0.001	0.03 ± 0.09	<0.001	0.931
UNVA (logMAR)					
Pre-op	0.77 ± 0.17	—	0.79 ± 0.16	—	0.439
1 week post-op	0.50 ± 0.16	<0.001	0.54 ± 0.16	<0.001	0.032
1 month post-op	0.50 ± 0.15	<0.001	0.51 ± 0.15	<0.001	0.518
3 month post-op	0.50 ± 0.13	<0.001	0.52 ± 0.15	<0.001	0.501
CNVA (logMAR)					
Pre-op	0.64 ± 0.16	—	0.65 ± 0.16	—	0.497
1 week post-op	0.17 ± 0.13	<0.001	0.24 ± 0.13	<0.001	<0.001
1 month post-op	0.17 ± 0.10	<0.001	0.18 ± 0.08	<0.001	0.509
3 month post-op	0.17 ± 0.09	<0.001	0.18 ± 0.10	<0.001	0.386
TCA (D)					
Pre-op	1.22 ± 0.46	—	1.17 ± 0.51	—	0.420
1 week post-op	1.29 ± 0.45	0.110	1.21 ± 0.48	0.399	0.211
1 month post-op	0.94 ± 0.47	<0.001	0.90 ± 0.46	<0.001	0.454
3 month post-op	0.94 ± 0.45	<0.001	0.92 ± 0.45	<0.001	0.733
SIA (D)					
3 month post-op	0.95 ± 0.44		1.01 ± 0.50		0.363
AE (°)					
3 month post-op	39.45 ± 26.53		49.75 ± 26.23		0.005

UDVA = uncorrected distance visual acuity; CDVA = corrected distance visual acuity; UNVA = uncorrected near visual acuity; CNVA = corrected near visual acuity; TCA = total corneal astigmatism; SIA = surgically induced astigmatism; AE = angle of error; *p*^a^ = comparison between pre-op and post-op and *p*^a^ < 0.05 was considered statistically significant; *p*^b^ = comparison of both groups and *p*^b^ < 0.05 was considered statistically significant.

**Table 4:** 10 mm CV and corneal endothelial cells.

	Steep-axis one-handed	*p* ^a^	Steep-axis two-handed	*p* ^a^	*p* ^b^
10-mm CV (*μ*m^3^)					
Pre-op	60.99 ± 3.37	—	60.61 ± 3.07	—	0.396
1 week post-op	63.59 ± 3.91	<0.001	65.06 ± 3.60	<0.001	0.005
1 month post-op	61.66 ± 3.43	0.011	62.04 ± 3.87	<0.001	0.467
3 month post-op	60.81 ± 3.09	0.437	60.66 ± 3.56	0.826	0.736
ECD (mm^2^)					
Pre-op	2539.91 ± 230.75	—	2570.21 ± 255.91	—	0.370
1 week post-op	2356.35 ± 238.08	<0.001	2349.93 ± 261.97	<0.001	0.853
1 month post-op	2350.37 ± 227.55	<0.001	2343.32 ± 252.55	<0.001	0.833
3 month post-op	2349.28 ± 229.39	<0.001	2342.31 ± 259.41	<0.001	0.837
ECL (%)					
1 week post-op	7.23 ± 3.91	—	8.58 ± 4.09	—	0.015
1 month post-op	7.40 ± 4.52	—	8.77 ± 4.99	—	0.039
3 month post-op	7.44 ± 4.70	—	8.83 ± 5.19	—	0.044
HEX%					
Pre-op	40.31 ± 6.57	—	40.04 ± 6.20	—	0.761
1 week post-op	39.48 ± 6.49	<0.001	39.35 ± 6.04	<0.001	0.883
1 month post-op	39.18 ± 6.00	<0.001	38.85 ± 6.26	<0.001	0.693
3 month post-op	39.03 ± 6.20	0.002	38.82 ± 6.85	<0.001	0.817

CV = corneal volume; ECD = endothelial corneal density; ECL = endothelial cell loss; HEX% = percentages of hexagonal cell; *p*^a^ = comparison between pre-op and post-op and *p*^a^ < 0.05 was considered statistically significant; *p*^b^ = comparison of both groups and *p*^b^ < 0.05 was considered statistically significant.

## Data Availability

The data used to support the findings of this study are available from the corresponding author upon request.

## References

[1] Borasio E., Mehta J. S., Maurino V. (2006). Torque and flattening effects of clear corneal temporal and on-axis incisions for phacoemulsification. *Journal of Cataract & Refractive Surgery*.

[2] Nemeth G., Berta A., Szalai E., Hassan Z., Modis L. (2014). Analysis of surgically induced astigmatism on the posterior surface of the cornea. *Journal of Refractive Surgery*.

[3] Khokhar S., Lohiya P., Murugiesan V., Panda A. (2006). Corneal astigmatism correction with opposite clear corneal incisions or single clear corneal incision: comparative analysis. *Journal of Cataract & Refractive Surgery*.

[4] Chiam P. J. (2015). Effect of paired opposite clear corneal incisions on with-the-rule versus against-the-rule astigmatism. *Cornea*.

[5] Kaufmann C., Peter J., Ooi K., Phipps S., Cooper P., Goggin M. (2005). Limbal relaxing incisions versus on-axis incisions to reduce corneal astigmatism at the time of cataract surgery. *Journal of Cataract & Refractive Surgery*.

[6] Zhang L., Sy M. E., Mai H., Yu F., Hamilton D. R. (2015). Effect of posterior corneal astigmatism on refractive outcomes after toric intraocular lens implantation. *Journal of Cataract & Refractive Surgery*.

[7] Slade S., Lane S., Solomon K. (2018). Clinical outcomes using a novel image-guided planning system in patients with cataract and IOL implantation. *Journal of Refractive Surgery*.

[8] Li P., Wu J., Guan Y. (2019). Comparative analysis of one-handed and two-handed coaxial phacoemulsification with 2.4-mm clear corneal incision. *Current Eye Research*.

[9] Li P., Zhang Y., Kang L., Guan Y., Wu J., Guan H. (2018). Comparison of variations in cornea after one-handed and two-handed coaxial phacoemulsification. *Clinical Ophthalmology*.

[10] Chylack L. T., Wolfe J. K., Singer D. M. (1993). The lens opacities classification system III. *Archives of Ophthalmology*.

[11] Alpins N. A. (1997). Vector analysis of astigmatism changes by flattening, steepening, and torque. *Journal of Cataract & Refractive Surgery*.

[12] Kawahara A., Kurosaka D., Yoshida A. (2013). Comparison of surgically induced astigmatism between one-handed and two-handed cataract surgery techniques. *Clinical Ophthalmology*.

[13] Masket S., Wang L., Belani S. (2009). Induced astigmatism with 2.2- and 3.0-mm coaxial phacoemulsification incisions. *Journal of Refractive Surgery*.

[14] Hashemi H., Khabazkhoob M., Soroush S., Shariati R., Miraftab M., Yekta A. (2016). The location of incision in cataract surgery and its impact on induced astigmatism. *Current Opinion in Ophthalmology*.

[15] Cavallini G. M., Campi L., Masini C., Pelloni S., Pupino A. (2007). Bimanual microphacoemulsification versus coaxial miniphacoemulsification: prospective study. *Journal of Cataract & Refractive Surgery*.

[16] Koç M., İlhan Ç., Koban Y., Özülken K., Durukan İ., Yılmazbaş P. (2016). Effect of corneal biomechanical properties on surgically-induced astigmatism and higher-order aberrations after cataract surgery. *Arquivos Brasileiros de Oftalmologia*.

[17] Woo S. J., Lee J.-H. (2003). Effect of central corneal thickness on surgically induced astigmatism in cataract surgery. *Journal of Cataract & Refractive Surgery*.

[18] Theodoulidou S., Asproudis I., Kalogeropoulos C., Athanasiadis A., Aspiotis M. (2016). Corneal diameter as a factor influencing corneal astigmatism after cataract surgery. *Cornea*.

[19] Yoon C. H., Kim M. K. (2018). Improving the toric intraocular lens calculation by considering posterior corneal astigmatism and surgically-induced corneal astigmatism. *Korean Journal of Ophthalmology*.

[20] Calabuig-Goena M., López-Miguel A., Marqués-Fernández V., Coco-Martín M. B., Iglesias-Cortiñas D., Maldonado M. J. (2016). Early changes in corneal epithelial thickness after cataract surgery—pilot study. *Current Eye Research*.

[21] Walkow T., Anders N., Klebe S. (2000). Endothelial cell loss after phacoemulsification: relation to preoperative and intraoperative parameters. *Journal of Cataract & Refractive Surgery*.

